# Thermal Tolerance and Preferred Temperature in the Critical Endangered Montseny Brook Newt (*Calotriton arnoldi*)

**DOI:** 10.3390/ani14131963

**Published:** 2024-07-02

**Authors:** Jenifer Contreras, Joan Gomà, David Velalcázar, Albert Montori

**Affiliations:** 1 Escuela de Ciencias Biológicas, Pontificia Universidad Católica del Ecuador, Avenida 12 de Octubre 1076, Quito 170143, Ecuador; jennifer.contreras92@hotmail.com; 2 Departament de Biologia Evolutiva, Ecologia i Ciències Ambientals, University of Barcelona, 08028 Barcelona, Spain; jgoma@ub.edu; 3 Freshwater Ecology, Hydrology, and Management Laboratory (FEHM-Lab), University of Barcelona, 08028 Barcelona, Spain; 4 Facultad de Ciencias de la Salud, Pontificia Universidad Católica del Ecuador, Av. Manuelita Sáenz, Ambato 180207, Ecuador; fdavid.velalcazar@gmail.com; 5 CREAC, Centre de Recerca i Educació Ambiental de Calafell, Secció Herpetologia, Aj, Calafell, 43882 Tarragona, Spain

**Keywords:** *Calotriton arnoldi*, critical thermal maximum (CTmax), thermopreference, thermopreferendum, temperature, thermal tolerance

## Abstract

**Simple Summary:**

Thermal tolerance and preferred temperature experimental procedures were conducted on the western and eastern subspecies of critically endangered Montseny Brook newt (*C. arnoldi*). The results obtained showed that the CTmax of the species exceeded 31 °C, with a significant difference between the two subspecies. We found that the species tolerates low temperatures (<1 °C) well. Although the thermopreference of the species was expected to trend to cold temperatures, some individuals chose relatively high temperatures, obtaining a range of 11.7 °C to 21.6 °C.

**Abstract:**

Climate change, driven by increased human greenhouse gas emissions since the beginning of the industrial revolution up to the present day, is considered one of the major threats to biodiversity in the twenty-first century. One of the most affected groups is the ectotherms due to their direct dependence on environmental temperatures. In recent years, several studies have analysed the effects of temperature and thermal tolerance on several species of ectotherms. However, there are species whose thermal tolerances are still unknown. Such is the case of the critically endangered species, the Montseny Brook Newt (*Calotriton arnoldi*), endemic to the Montseny massif in Spain and whose thermal biology is unknown. Its critical situation makes it essential to know its tolerance to cooling, warming and thermopreferendum in water environments where the newt lives. Three experimental procedures were conducted from the western and eastern subspecies of *C. arnoldi*, considering four classes separately (males, females, juveniles and larvae). The results obtained showed that the CTmax of the species exceeded 31 °C, with a significant difference between the two subspecies. We found that the species tolerates low temperatures (<1 °C) well because the genera *Calotriton* is adapted to live in cold waters with temperatures below 15 °C. Although the thermopreference of the species was expected to trend to cold temperatures, some individuals chose relatively high temperatures, obtaining a range of 11.7 °C to 21.6 °C. The results presented in this study are an advance in the knowledge of the thermal physiology of this species and support the importance of the temperature of the torrent on its survival. Knowing their thermal limits and their preferred temperature range will help to propose management measures that promote the conservation of streams and riparian forest cover to mitigate temperature increases due to climate change.

## 1. Introduction

Climate change, driven by increased human greenhouse gas emissions since the beginning of the industrial revolution up to the present day, is now considered one of the major threats to biodiversity in the twenty-first century and one that underlies several reported extinction events [1,2,3]. This is due to the rapid increase in Earth’s temperature in recent decades [1]. Throughout evolutionary history, species have shown resilience to certain temperature increases. However, the current rate of temperature rise poses a threat to the rapid adaptation of organisms, jeopardising their survival [4,5]. Consequently, numerous studies have been conducted in recent decades to demonstrate the effects of temperature changes on various organism groups [6,7,8,9,10,11,12]. Ectotherms are among the groups most affected by global warming [13,14], as temperature impacts their organisation at all levels, from cellular to enzymatic processes [15,16,17,18,19]. These processes determine certain traits, among which are growth, reproduction, metabolism, parasitic relationships, survival and distribution range [11,15,16,17,18,19,20].

The study of thermal ranges is essential to understanding numerous aspects of the biology of organisms since they represent the conditions that limit their fundamental niche and, therefore, their presence and evolution in a certain habitat or geographical area [11,15,21,22,23]. The thermal zone between upper and lower critical limits shapes the thermal tolerance range, where the given species can tolerate the environmental temperatures. The susceptibility of a population, species or community to receive a negative impact due to climate change will depend on a combination of two factors: First, the sensitivity of the organisms, controlled by intrinsic factors such as thermal tolerance limits (CTmax and CTmin). Second, the amount and variation in exposure to extrinsic environmental stressors, such as extreme temperatures due to climate change [6,7,8,9,10,11,12]. The most recent inferences regarding the impacts of global warming on biodiversity have focused mainly on the extrinsic factors that potentially determine the distribution of species in front of different climate change scenarios [11,19,20,24]. Ectotherms can experience large variations in body temperature during their life cycle or even during a single day, and physiological functions are considerably modified depending on thermic scenarios.

Frost tolerance is one of the best examples of how amphibian species adapt and survive in harsh thermal environments. Two main principles of animal frost tolerance have received much attention: Many amphibians seek refuge from freezing temperatures with the simplest solution for protection: moving underwater or underground for the winter months to place themselves where they have almost no chance of experiencing extreme freezing temperatures [25]. Other amphibians promote the production of high concentrations of organic osmolytes (glucose, glycerol, urea in amphibians) that protect the intracellular environment and control ice formation in the body [26,27]. In these harsh conditions, these species are at high risk of death from dehydration or freezing. [25,26,27]. The Siberian Salamander (*Salamandrella keyserlingii*) [25] tolerates winter temperatures around −50 °C. This salamander has no physiological protection against desiccation or freezing and its survival during freezing is entirely determined by the characteristics of the substrate. However, other species such as *Rana arvalis* [26] synthesise glucose and glycerol as cryoprotectants, and *R. sylvatica* from subarctic populations are able to survive freezing by accumulating urea and storing glycogen from which glucose is mobilised during freezing [27,28]. In temperate latitudes, efficient cryoprotective mechanisms have also been described in some species, such as *Rana temporaria* and *Bufo bufo* [29,30] and *Pelophylax esculentus* and *P. lessonae* [31]. 

However, most amphibian species live in warmer areas and do not have these metabolic adaptations to prevent freezing death during non-existent wintering, although similar mechanisms have been described to overcome warming seasons and prevent dehydration [32]. Surprisingly, studies on the thermal physiology, thermal behaviour and requirements of semi-aquatic amphibians, such as newt species, remain largely unexplored [33].

Some newts, such as species of the genus *Calotriton*, are adapted to live in streams with cold and running waters, in the middle of high mountains with well-oxygenated water due to the absence of or functional reduction in their lungs. These species do not inhabit aquatic habitats where the water temperature exceeds 16 °C or drops below 4 °C. However, while *Calotriton asper* spends almost its entire life cycle in the aquatic environment and could be considered a semi-aquatic amphibian, *C. arnoldi* is a strictly aquatic species, mainly occupying the subterranean aquifer of streams. These differences are a consequence of the wide environmental thermic range in which *C. arnoldi* lives and the location of the species in habitats with a strong Mediterranean climate influence [34], where freezing temperatures are unlikely to be reached. These two factors do not allow terrestrial movements of the species or immatures as occurs in the sister species, which winter on land, and restrict the possibilities of behavioural regulation of body temperature.

Given the significance of temperature in the biology and phenology of ectotherms, it is crucial to understand the thermal physiology of organisms for which thermal physiology is unknown, such as the Montseny Brook Newt (*Calotriton arnoldi*). Moreover, this species is listed as critically endangered (CR), according to the IUCN [35]; it exhibits a limited geographical distribution, and the current population trend is decreasing. Until now, two morphologically and genetically distinct subpopulations have been identified here, the eastern and western subpopulations, with no gene flow between them [34,35,36,37,38]. However, a recent study [39] described the Western Montseny Brook Newt as a new subspecies: *Calotriton arnoldi laietanus*. Under this new scenario, the two commonly known subpopulations are distinct evolutionary processes, which have formal subspecific designation [39]: eastern populations as *Calotriton arnoldi arnoldi* and western populations as *C. a. laietanus*. These authors [39] reanalysed published morphological data to delve into their phenotypical differentiation and, through the integration of genomic, morphological and ecological evidence of the distinctiveness of both lineages, provide clear evidence of the differences in habitat, thermal, abiotic and biotic conditions of the habitat occupied by the two subspecies. In addition, the survival of the species has been primarily affected by logging, deforestation [37] and global warming [40]. Given the known relationship between temperature, climate trends and the diversity of responses and strategies of the species of salamanders and newts [17,18,40,41,42,43], it is imperative to understand how *C. arnoldi* responds to temperature variations, a study not conducted until now, to propose management measures to minimise the effects of this thermal increase. Therefore, the aims of this study were to (1) determine the critical thermal maximum (CTmax), (2) determine the tolerance of individuals to cold temperatures around 1 °C (3) determine the preferred body temperature for *C. arnoldi* and (4) hypothesise about the future of the populations in a climate change scenario.

## 2. Materials and Methods

### 2.1. Biosecurity Standards Used

Strict safety measures were employed for the experiments due to the endangered status of the species. Everybody in contact with the newts had to disinfect their hands and wear surgical gloves [44,45]. All laboratory instruments and materials in contact with water and newts were disinfected with alcohol or sanitiser and rinsed with water before each experiment and between each group of individuals. Water was changed between each group of individuals and between both subspecies. Each group of individuals undergoing experiments was observed by more than one researcher to analyse signals, determine the end of the experiments and prevent any incidents. None of the 60 individuals used for the experiments suffered any harm, and all were returned to their aquariums at the end of the experimentation.

### 2.2. Experimental Procedure

The study involved specimens exclusively of *C. arnoldi* kept at the Wildlife Recovery Center of Torreferrussa, located in Santa Perpetua de la Mogoda, Barcelona, Spain. These newts are maintained in aquariums with temperatures ranging from 9 °C in winter to 15 °C in summer, with lights cycling on and off to simulate natural photoperiods. The two subspecies, eastern and western, are kept separate within the centre to maintain genetic isolation. For this reason, the three experiments (CTmax, CTmin and thermopreferendum) were conducted at different times, first with the western subspecies and then with the eastern subspecies. The experimental procedure was conducted from April to July 2019. A total of 30 individuals from each subspecies, divided into 5 different groups (6 adult males, 6 adult females, 6 metamorphic juveniles, 6 larvae born in 2017 and 6 larvae born in 2018), were selected for the experiments. Each group underwent the three experiments detailed in Section 2.2.1, Section 2.2.2 and Section 2.2.3.

#### 2.2.1. Critical Thermal Maximum (CTmax)

The experiment was conducted following a similar procedure to [15,21,46]. Before the experiment, the 60 individuals from both subspecies of *C. arnoldi* were acclimated to an average temperature of 11.3 °C. Each individual was weighed (Supplementary Materials Table S1) and then placed in a metal mesh basket measuring 8.5 cm in diameter × 8 cm in depth, with a plastic mesh lid to prevent newts from escaping. The 6 baskets were placed in a steel tank measuring 21 cm wide × 51 cm long × 45 cm deep, containing 18 L of water from the aquariums and thus at the newts’ acclimation temperature.

**Figure 1:**
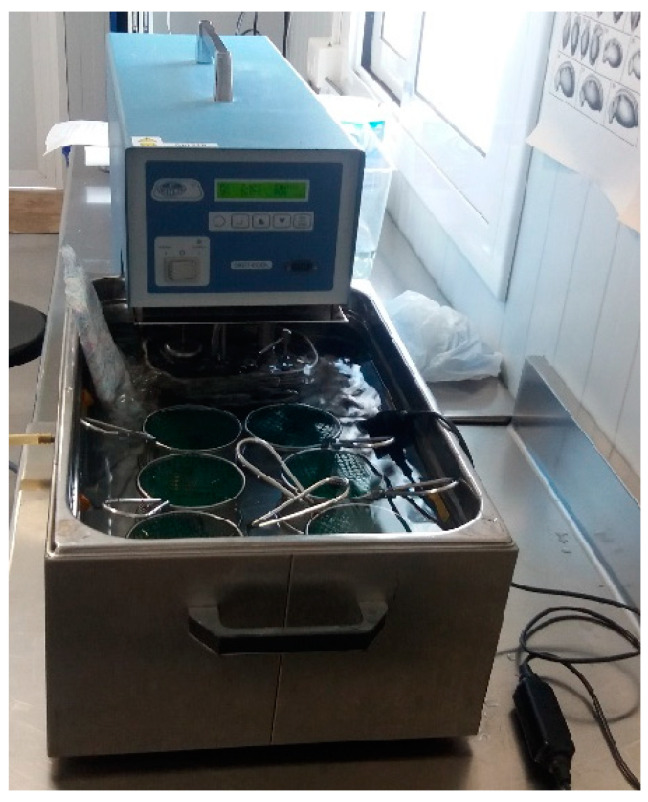
Experimental design used (steel tank, baskets and digit cool thermostat) to measure the CTmax and cold tolerance of *Calotriton arnoldi*.

A “Digit Cool” immersion thermostat from J.P. Selecta^®^ (Barcelona, Spain) was used to heat the water. The temperature was increased by 1 °C (±0.5 °C) every 3 min, and a digital HQ40D multimeter from Hach^®^ (Düsseldorf, Germany) was used to measure the temperature (Figure 1). The endpoint of the experiment was the same as that considered by other authors: a set of continuous spasms, loss of locomotion or response to stimuli and loss of righting response, following the technical procedure of [46]. To determine the response to stimuli, a gentle rubber stirrer was used to lightly touch the individuals and observe their reactions.

#### 2.2.2. Cold Tolerance

The procedure was conducted using the same methodology used in the heat experiment, with the same number of individuals per subspecies. The “Digit cool” immersion thermostat from J.P. Selecta^®^ was used to cool the tank water, decreasing the temperature by 1 °C (±0.4 °C) every 3 min. The water temperature was recorded using the HQ40D digital multimeter from Hach^®^. The endpoint of the experiment was set at 1 °C due to issues with the cooler, preventing testing temperatures below zero and determining the minimum thermal tolerance. Each observation and the response of the newts to stimuli were recorded as in the previous experiment. In strictly aquatic urodela such as *C. arnoldi*, which uses cold streams with minimum water temperatures around 4 °C, located in temperate regions, water does not reach freezing temperatures, and the wintering period occurs in underground aquifers where the water temperature is buffered.

#### 2.2.3. Preferred Temperature (Tp)

**Figure 2:**
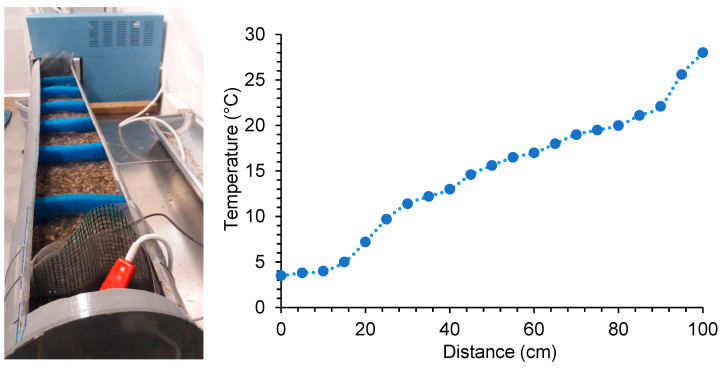
(**Left**): Thermopreferendum channel used to measure the preferred body temperature of *Calotriton arnoldi*. (**Right**): Temperature gradient obtained in the 1 m thermopreferendum channel. The minimum temperature was 3.5 °C, and the maximum temperature was 28 °C.

To perform this experiment, a temperature gradient was created in water using a PVC pipe channel measuring 1.50 m in length and 20 cm in diameter. The tube was divided into three sections separated by fine metal mesh discs. The first section, 25 cm long, was where a cold source was placed (the same immersion thermostat used in previous experiments), maintaining the temperature at 3.5 °C. The second section, 1 m long, formed the temperature gradient; at the bottom of this section, approximately 10 cm of gravel was placed, and above it, 2 cm of water from the aquariums. The third section, 25 cm long, had a heat source (water heater) connected to a thermostat, maintaining the temperature at that end at 28 °C. Sponges were placed inside the channel every 10 cm, with a hole for newts to pass through; the sponges prevented convective water movement, forming a more stable gradient (Figure 2). It is essential to emphasise that for the gradient to function correctly, constant heat and cold sources, a surface maintaining stable temperatures in each zone (in this case, gravel), and elements preventing excessive water circulation within the channel (e.g., sponges) are necessary. Once the gradient was formed, the temperature was measured every 5 cm using a fast-response K thermometer from HIBOK 14. The gradient ranged from 3.5 °C to 28 °C, with an approximately 1 °C increase every 5 cm (Figure 2). Only 6 males, 6 females and 6 juveniles from both subspecies were used for this experiment. The newts were placed in groups of 3 individuals, each group positioned in the channel at the temperature of their acclimation and observed for 40 min. After this time, the temperature of the location where the individual was found, the body temperature, the length from snout to cloaca and the weight were recorded. After each experiment, the temperature was measured every 5 cm in the channel to observe if the gradient remained stable.

### 2.3. Statistical Analysis

For the statistical analysis of maximum critical temperature (MCT) and thermopreferendum (Tp), the “Car” [47], “Stats” [48] and “Pgirmess” [49] packages in the R studio programme (version 1.2.1335) were used. For CTmax and cold tolerance, average values of weights, critical thermal maximum and minimum reached were calculated for each group of individuals from both subspecies. Subsequently, correlation tests were performed to determine whether there was a correlation between CTmax and the weights of all individuals. Pearson correlation was used for groups with normally distributed variables, and Spearman’s non-parametric correlation was used for groups in which one or both variables did not show normality. The analyses of CTmax for each group were first performed with both subspecies combined and then for each subspecies individually. For parametric data, where the Levene test was not significant (*p* > 0.05), one-way ANOVAs and post hoc Tukey tests were used. For non-parametric data, where the Levene test was significant (*p* < 0.05), a Kruskal–Wallis test with its respective post hoc multiple comparison Bonferroni test was used. After analysing each subspecies, a pairwise test was conducted between each group of individuals from the two subspecies. Due to the lack of normality in the data, a Wilcoxon paired test was used for comparisons.

For the analysis of data obtained in the thermopreferendum experiment, averages of the temperature at the end of the experiment (Tp), as well as the weight and snout–vent length (SVL) for each group of individuals, were obtained. To determine the preferred temperature range for each group, the tenth and ninetieth percentiles were calculated, which previous studies reliably characterise as the thermopreferendum range in newts [16,17,18]. Similar to the CTmax analyses, a series of correlations were performed. Pearson correlations were used to determine whether there was a relationship between Tp and the weight of individuals and between Tp and their SVL. Subsequently, an analysis of Tp for the entire species, i.e., both subspecies combined, was performed using a one-factor ANOVA with Tukey’s post hoc test. Next, the analysis was conducted for each population; for the eastern population, the same statistics used for the entire species were employed, and for the western population, a Kruskal–Wallis test was used. Finally, paired *t*-tests were conducted between the groups of both subspecies to observe whether there were differences between them.

## 3. Results

### 3.1. Critical Thermal Maximum (CTmax)

At the end of the experiment, all individuals showed a low or no response to external stimuli. The thermal limit was characterised by limb spasms, body contractions, stiffness and, ultimately, complete paralysis, from which they immediately recovered upon contact with water at a lower temperature.

Upon analysing both subspecies together, it was determined that the CTmax was 31.7 °C (±0.04) for males (N = 12), 31.42 °C (±0.05) for females (N = 12), 31.53 °C (±0.15) for juveniles (N = 12), 32.51 °C (±0.32); for larvae born in 2017 (N = 12) and 31.69 °C (±0.19) for larvae born in 2018 (N = 12). In Table 1, we show the results for the two subspecies (eastern and western) separately. Upon analysing the correlations between weight and TCmax, it was observed that none were significant (*p* > 0.05), indicating that in none of the groups, CTmax is related to the weight of the individuals (Table 1).

**Figure 3:**
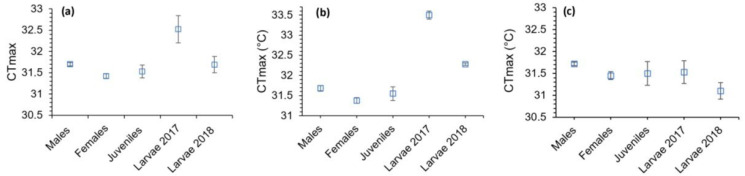
Average critical thermal maxima (CTmax) of the five groups of *Calotriton arnoldi*: (**a**) both subspecies, (**b**) western subspecies (*C. a. laietanus*) and (**c**) eastern subspecies (*C. a. arnoldi*). Bars represent standard error.

When performing the Kruskal–Wallis test for the entire species, highly significant differences were observed between the groups (χ^2^ = 13.43; *p* < 0.01). The post hoc Bonferroni test of multiple comparisons determined that the CTmax of females and larvae born in the year 2017 varied significantly (*p* < 0.05) (Figure 3a). Subsequently, when analysing the subspecies separately in the western population, it was determined through the non-parametric Kruskal–Wallis test that there were significant differences between the groups (χ^2^ = 23.85; *p* < 0.0001). Differences were found between the CTmax of females with both groups of larvae from 2017 and 2018, between larvae from 2017 and males and between larvae from 2017 and juveniles (*p* < 0.05) (Figure 3b). For the eastern population, the result of the ANOVA was not significant (ANOVA: F = 1.39; *p* > 0.05), indicating that there were no significant differences between the CTmax of the five groups in this population (Figure 3c).

Statistical analyses obtained by making paired comparisons between the two subspecies indicated that only the larvae from 2017 and 2018 showed significant differences between the subspecies (W = 36; *p* < 0.01 in both cases). The rest of the groups, males, females and juveniles, did not have significant differences in Ctmax between the two subspecies.

Additionally, throughout the experiment, certain signs indicated that individuals were no longer within their comfort temperature. These signs included vomiting, head movement from side to side, small spasms and mouth opening. Given the similarity in Ctmax between males, females and juveniles from the two subspecies, the temperature at which these discomfort signals occurred was considered a single group. Notably, out of the total individuals, 13 exhibited these discomfort signals at an average temperature of 26.24 °C (±0.27), with a minimum of 24.5 °C and a maximum of 27.3 °C.

### 3.2. Cold Tolerance

At the end of the experimentation with low temperatures, it was observed that individuals from the western population, including males (N = 6), larvae from 2017 (N = 6) and larvae from 2018 (N = 6), resisted without reaching their critical minimum temperature up to 1 °C. However, in females (N = 6), two individuals did not withstand this temperature and had to be removed from the experiment at 1.1 °C and 1.5 °C, respectively. Similarly, in juveniles from the same population (N = 6), this behaviour was observed in three individuals (two at 1.1 °C and one at 1.2 °C). This indicates that out of 30 individuals from the population across the five groups, only 5 individuals reached the Ctmin at an average temperature of 1.2 °C (±0.08). The signals exhibited by these individuals were similar to those observed for Ctmax. For the remaining individuals, the temperature at which they became lethargic and responded slowly to external stimuli was determined, averaging 2.05 °C (±0.19). In the eastern population, it was observed that all individuals from the five groups, males, females, juveniles, larvae from 2017 and larvae from 2018, could withstand temperatures up to 1 °C without reaching their Ctmin. The lethargy temperature for these individuals was determined to be 1.78 °C (±0.18).

### 3.3. Preferred Temperature (Thermopreferendum-Tp)

The newts used in the experiment exhibited similar behaviour across all groups. When placed in the thermopreferendum channel (Tp), they actively moved along the gradient for several minutes until they became stationary in the zone they preferred. After approximately 20 min, they showed reduced activity in the channel. Notably, no individual moved to the last box ranging from 22 to 28 °C, nor towards the opposite end where the temperature ranged from 3.5 to 5 °C.

In the overall analysis of the entire species, both subspecies combined, males (N = 12) had a Tp of 14.89 °C (±0.92) within a range of 11.7 to 19.48 °C. Females (N = 12) exhibited a Tp of 17.51 °C (±0.67) within a range of 15.22 to 20.88 °C. Finally, juveniles (N = 12) showed an average Tp of 18.24 °C (±0.87) within a range of 13.79 to 21.5 °C. In Table 2, we show the results for the two subspecies (eastern and western) separately. Correlations between Tp and individual weight, as well as Tp and body length, were not significant (*p* > 0.05), indicating that Tp for different groups was not related to these variables (Table 2).

The ANOVA conducted for the data obtained for the entire species had a significant result (F = 4.55; *p* < 0.05), indicating differences among the three groups (males, females and juveniles). Post hoc Tukey test determined that the only significant difference was between males and juveniles (*p* < 0.05).

**Figure 4:**
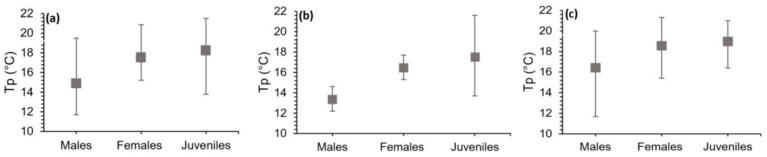
Preferred body temperature (Tp) of the three groups of *Calotriton arnoldi*: (**a**) both subspecies, (**b**) western subspecies (*C. a. laietanus*) and (**c**) eastern subspecies (*C. a. arnoldi*). Bars represent the thermopreferendum range of each group.

Males exhibited a lower Tp and a temperature range that included relatively colder temperatures (Figure 4a). When analysing the results for each population separately, significant differences were observed between groups in the western population (χ^2^ = 8.80; *p* < 0.05), specifically between males and females and males and juveniles (*p* < 0.05). This result clearly indicates that males have a preferred range with lower temperatures than the other two groups. Additionally, juveniles can occupy a broad range of temperatures that even surpass those of the adults (Figure 4b). For the eastern population, the ANOVA did not reveal significant differences among the three groups (F = 1.17; *p* > 0.05). However, it can be observed that males still exhibit a lower Tp, as observed in the other population, although not statistically proven (Figure 4c). When performing pairwise comparisons using the *t*-test, no significant differences were found between males, females and juveniles from the two subspecies (*p* > 0.05).

## 4. Discussion

### 4.1. Thermal Tolerance of Calotriton arnoldi Species

The critical thermal maximum (CTmax) is determined by a set of spasms and loss of locomotor response [21,46]. Considering this background, it can be stated that individuals of *C. arnoldi* behaved similarly to what [15,21,46] described. In other words, most individuals had a well-defined thermal limit from which they recovered upon contact with cooler water. Considering both subspecies of *C. arnoldi* together, all groups present a similar average critical thermal maximum (CTmax), surpassing 31 °C, except the larvae born in 2017, who showed a significant difference from the other classes (Figure 3). However, this occurred only in the western subspecies. Both 2018 and 2017 larvae had significantly higher CTmax compared with the other groups.

One possible hypothesis to explain these significant differences in CTmax only in the western population could be related to the dissimilarities in habitat structure [37,38,39] and temperature range in western streams. In fact, the maximal and minimal water temperatures of western subspecies streams are slightly higher than those of eastern streams [37,38,39] (mean east: 11.6 °C; mean west: 13.7 °C). The Western and Eastern *Calotriton arnoldi* subspecies inhabit ranges that significantly differ in altitude, temperature and precipitation regimes and are associated with dissimilar forest assemblages [39]. These differences between subspecies could have led to the development of some local temperature adaptation factor during the larval stage, given the morphological and genetic differences between the two subspecies [36,39]. The divergence between Eastern and Western Montseny lineages seems to have taken place later, around 111 kya (68–155 kya), roughly coinciding with a warmer period: the Riss-Würm Interglacial [38,39]. 

Previous studies have shown that differences in CTmax within a species or between several species of salamanders are correlated with habitat thermal regimes. Therefore, optimal CTmax adjustment patterns can vary from one habitat to another [15,41]. This ecological adaptation or perhaps plasticity in thermal adjustment needs to be studied in detail because, in other stream-dwelling newts [50], there have been identified significant intraspecific morphological and niche differentiation, suggesting that newt morphology—or CTmax in our study—is responsive to environmental factors. Fluctuations in temperature and the relationship between physiological maxima and environmental climate could be geographically variable and dependent on extrinsic more than intrinsic characteristics [51]. However, the lack of knowledge of the thermal ecology of *C. arnoldi* and its physiology does not allow us to define what factors determine these differences and whether they are adaptive with a genetic basis or a result of phenotypic plasticity or acclimatisation.

In western subspecies, we also found significant differences in CTmax between the larvae born in 2017 and 2018. We hypothesise that the age of the larvae could be the explanation for this dissimilarity. Some studies in different amphibian species have demonstrated differences in CTmax between larvae at different stages and adults due to thermal pressures. However, the reason for these differences is poorly understood [52,53]. In the case of this study, it could be that the 2017 larvae have already lived 1 year in water with high and low water temperatures in summer and winter, respectively, and, therefore, larvae born in 2017 may have acquired some thermal tolerance or acclimation compared with the young 2018 larvae. 

Despite these differences between larvae, the species showed a CTmax ranging between 31 and 33 °C. This range is like other species of salamanders in temperate zones, which have CTmax ranges from 32 to 34 °C [15]. Additionally, during the study, 13 individuals showed significant signs of discomfort, one of them being vomiting at an average temperature of 26 °C (24.5 °C–27.3 °C). These signs could indicate the thermal conformity limit for the species, which, if prolonged, could be detrimental to its survival, as has been observed in other larval species [53], in which it was found that response to warm water for extended periods may be lethal. This temperature could be more plausible to reach in nature because in western streams, some records of water temperatures exceed 20 °C slightly [37], and in the future, these values will be more frequent and prolonged in time in the context of climate change [40].

On the other hand, tolerance to cold or freezing is a key factor in the survival of organisms. It has been observed that some ectotherms, mostly in temperate or subarctic zones, exhibit resistance to extremely low temperatures, even below freezing [25,27,54,55]. Therefore, cold tolerance tends to be extreme or higher in temperate zones, where latitude is greater. Altitude also plays a significant role, with species from higher altitudes being more resistant than those from lower altitudes [56]. As *C. arnoldi* is a species that mainly inhabits cold-water streams in Montseny [57,58] and also thrives in elevated areas, it was expected to have a fairly high cold tolerance, as demonstrated in this study.

During the experimentation, it was observed that most individuals began to become sluggish and move slowly just before or after reaching 2 °C. However, they did not show any signs of having reached their critical thermal minimum (CTmin), indicating that their limit would be at temperatures below 1 °C. This tolerance has been described in other amphibians such as *Desmognathus fuscus*, a salamander inhabiting mountain streams, which can exhibit a CTmin below 0 °C depending on acclimatisation [41]. There is a known relationship between both critical temperatures, and the hypothesis suggests that maintaining a low CTmin can be costly for organisms with a high CTmax and vice versa [41,56]. This could explain why *C. arnoldi* presents good cold tolerance, but its CTmax is not as high as that of other tropical organisms, which may have CTmax exceeding 40 °C [59]. However, as acclimatisation temperatures were not used in this study, it is suggested that future measurements of CTmax and cold tolerance consider this factor in determining whether there are variations.

### 4.2. Preferred Body Temperature Range (Tp) of Montseny Newt in the Laboratory

*Calotriton arnoldi* individuals are known to inhabit cold-water streams not exceeding 15 °C, with steep slopes and at an altitude ranging from 600 to 1200 m above sea level [34,37,57,58], with clear differences between the two subspecies [39]. However, the preferred body temperature range (Tp) of the species was unknown until this study. It was determined that this species exhibits a broad thermopreferendum range (under laboratory conditions), ranging from cold temperatures of 11.7 °C to moderate temperatures of 21.6 °C. This was unexpected, as it was initially believed that the species would prefer colder temperatures.

Although the Tp range presented by this species seems high, it is lower than the Tp range of the *Calotriton asper* sister species, which had a temperature range from 11.10 °C to 32 °C, depending on the altitude of the individuals [43]. These authors observed that *C. asper* individuals at higher altitudes had a broader Tp range than those at lower altitudes. This demonstrates that this species did not follow what is postulated in the “optimal local hypothesis”, which states that individuals at lower altitudes will have a higher Tp than individuals at higher altitudes [60]. On the contrary, they adhered to the “countergradient hypothesis”, which states that organisms at medium and high altitudes seek warmer preferred temperatures to compensate for the limited opportunity to be in favourable temperatures [61] and thus perform vital functions for their life cycle (locomotion, reproduction, digestion) [42]. This may explain to some extent why *C. arnoldi* individuals exhibited a thermopreferendum with temperatures often exceeding 15 °C or the temperature to which they were acclimated.

It is important to emphasise that there are differences in thermopreferendum among different groups (males, females and juveniles) within the species, both in the western and eastern subspecies. These differences primarily stem from the fact that males within the thermopreferendum channel choose colder temperatures than females and juveniles. Moreover, juveniles might be capable of occupying larger Tp ranges than adults, even reaching higher temperatures than the other two groups. These differences could be attributed to the fact that, in nature, males are found in colder and more oxygenated waters, while females and juveniles tend to be in warmer waters and concealed in the stream [62,63]. An important consideration for future studies is that thermopreferendum could increase or decrease due to various factors such as pregnancy in females, digestion [16,18], seasonality and acclimatisation [64]. In fact, for the sister species *Calotriton asper* [62], it was corroborated that they were mainly thermoconformers, but the body temperature of newts was slightly higher than water temperature in individuals in amplexus, and seasonally, females showed much greater dispersion in cloacal temperature than males, probably related to spawning behaviour. Previous studies have shown that acclimatisation did not influence the Tp range choice in some species in temperate zones [15,42]. Therefore, due to the current lack of knowledge about *C. arnoldi*, it is necessary to conduct a study that involves more factors such as seasonality, digestion and pregnancy, among other parameters, to determine whether there is an increase or decrease in Tp and body temperatures throughout the year in this species.

### 4.3. Ecological Implications and Management Measures

From the analysis of the data obtained in this study, we can observe the significant influence of temperature on the species *C. arnoldi*, which is crucial for a better understanding of its biology and ecology. Understanding the patterns that lead to the thermal physiology of a species is important to comprehend the evolutionary responses that different organisms may present to climate variations, thereby predicting their future and responses to climate change more effectively [65]. Temperature monitoring in Montseny determined that the ambient temperature is rapidly increasing, averaging 0.3 °C per decade in the second half of the 20th century [40]. This, combined with the deforestation suffered by the newt’s habitat [65,66] and the expansion of the oak forest towards beech forest natural areas, has led to prolonged drought periods that threaten the species’ survival in the wild [65].

While it is true that it is highly unlikely that temperatures in streams will increase to reach the species’ critical maximum temperature (CTmax) in the near future, there is a high probability their temperature may rise to levels unsuitable for the species or even that streams may dry up. This is due to the temperature increase in the massif, especially due to the loss of vegetation cover, as direct radiation significantly affects water temperature [65]. The suboptimal temperature for the species can be set at 26 °C, where individuals showed signs of discomfort, such as vomiting. Reaching this temperature or experiencing prolonged drought periods would have several implications for the species. On the one hand, it could alter its distribution range [11,20], as happened with its sister species *C. asper*, in which climate change reduced its range, and due to its limited dispersal capacity, genetic loss may have occurred [67]. This can also happen to the Montseny newt, which would have to migrate during warmer or even dry periods to find niches where stream temperatures are within its preferred or optimal range. If suitable sites are not found, individuals and genetic diversity may be lost, potentially leading to the species’ extinction [38,67]. This is particularly evident for the nominal subspecies *C. a. arnoldi*, which has a much smaller distribution and occupies smaller sections of streams than the western subspecies [39]. The streams currently occupied by the eastern subspecies are, in many cases, restricted to the upper parts of the basins, making it impossible to move up in altitude, as is currently happening in many Iberian amphibian species due to rising temperatures [11]. Additionally, the poor dispersal capacity of the species [37] compromises the future of the eastern subspecies.

Studies carried out between 1983 and 2015 in one stream located in the distribution area of *C. arnoldi* [68] showed that while the air temperature has increased by 0.6 ± 0.057 °C/decade, the water temperature has not changed. The authors proposed two hypotheses: groundwater-fed streams, especially during the dry season, and the maintenance of a high riparian canopy along the stream would avoid direct solar radiation [68]. However, drought periods are expected to become more frequent in the next decades [1]. The eastern streams, where *C. a. arnoldi* inhabits, commonly experience droughts during summer, resulting in the disappearance of most of the surface water. In contrast, surface water levels in the western streams, where *C. a. laietanus* inhabit, are more constant and have surface water throughout the year [39]. 

When considering the dire situation faced by this species in its natural habitat, especially the eastern subspecies, and taking into account the contributions from [69], it can be argued that one of the proposed management measures for the conservation of this species would be to maintain the vegetation cover along streams where newts are found. This is to prevent an increase in their temperature, as observed by [53], who reported that the absence of or reduction in forest canopy after logging results in increased stream temperatures. These effects may be amplified in areas with a variable Mediterranean climate like the Montseny massif, where streams have cold but low groundwater flows, making them more vulnerable to warming [24]. Additionally, water extraction from Montseny streams must be regulated by authorities. Excessive exploitation of this resource, which is essential for the newt, could lead to habitat loss or destruction. Implementing these measures could provide safe habitats for the development of existing species and subspecies plans for reintroduction [37,70,71].

## 5. Conclusions

During this study, it was concluded that *Calotriton arnoldi* has a CTmax that exceeds 31 °C in all groups. The larvae of the western population are the only ones that have a CTmax higher than 32 °C, which could be due to habitat heterogeneity and their developmental stage. On the other hand, males, females and juveniles did not show differences between the two subspecies. It was also determined that temperatures from 24.5 °C to 27 °C could be a conformity limit that should be studied thoroughly.

It was observed that the Montseny newt, being a species found in high-altitude areas and in cold-water streams, had a good cold tolerance in all studied groups. Its minimum critical temperature would likely be less than 1 °C.

It was also determined that the thermopreferendum of *C. arnoldi* ranged from 11.7 °C to 21.6 °C. It is a broad range but lower than the one reported for its sister species, *C. asper* (11.10–32 °C). However, it preferred warmer temperatures more than expected. This can be explained by the “countergradient hypothesis”. It was also observed that males chose colder temperatures more than females and juveniles in both subspecies (more in *C. arnoldi laietanus* than in *C. a. arnoldi*). This can be due to differences in the species’ ecology.

The results presented in this study advance knowledge of the thermal physiology of this species and support the importance of stream temperature for the survival of *C. arnoldi*. Knowing its thermal limits and its preferred temperature range will help to propose management measures that promote the conservation of Montseny streams and their riparian vegetation to mitigate temperature increases due to climate change.

## Figures and Tables

**Table 1:** Average weight, critical thermal maxima (CTmax), correlation between weight and CTmax in the five groups of the two subspecies of *Calotriton arnoldi*.

Western Subspecies (*C. a. laietanus*)							
	Weight (g)	CTmax (°C)	S.E	Min (°C)	Max (°C)	R^2^	*p*-Value
Males	5.70	31.68	0.07	31.4	31.8	−0.39	0.44
Females	7.03	31.38	0.07	31.1	31.5	−0.52	0.29
Juveniles	2.33	31.55	0.17	31.0	32.1	−0.26	0.61
Larvae 2017	0.75	33.50	0.10	33.0	33.6	0.67	0.14
Larvae 2018	-	32.28	0.03	32.2	32.4	-	-
**Eastern Subspecies (*C. a. arnoldi*)**							
Males	6.12	31.72	0.04	31.6	31.8	−0.74	0.09
Females	4.33	31.45	0.09	31.0	31.6	−0.51	0.30
Juveniles	2.35	31.50	0.27	30.2	31.9	−0.21	0.69
Larvae 2017	1.40	31.53	0.26	30.5	32.2	−0.30	0.56
Larvae 2018	-	31.10	0.19	30.5	31.5	-	-

**Table 2:** Average weight, body length, preferred temperature (Tp), temperature range, correlation between weight and Tp and correlation between SVL and Tp in the three groups of the two subspecies of *Calotriton arnoldi*.

Western Subspecies (*C. a. laietanus*)										
	Weight (g)	SVL (cm)	Tp (°C)	SE	Min	Max	R^2^_Weight-Tp_	*p*-Value	R^2^_SVL-Tp_	*p*-Value
Males	5.15	5.95	13.35	0.50	12.2	14.6	−0.40	0.42	−0.15	0.78
Females	4.73	6.13	16.45	0.48	15.3	17.7	−0.76	0.08	−0.01	0.98
Juveniles	2.08	4.48	17.51	1.46	13.7	21.6	0.68	0.13	0.76	0.08
**Eastern** **Subspecies (*C. a. arnoldi*)**										
Males	5.63	6.05	16.43	1.58	11.7	20.0	−0.07	0.89	0.56	0.24
Females	3.98	5.61	18.56	1.13	15.4	21.3	−0.29	0.57	−0.03	0.95
Juveniles	2.2	4.32	18.97	0.98	16.4	21.0	−0.12	0.82	−0.06	0.92

## Data Availability

The raw data supporting the conclusions of this article will be made available by the authors on request.

## Supplementary Materials

The following supporting information can be downloaded at: https://www.mdpi.com/article/10.3390/ani14131963/s1, Table S1: *C. arnoldi*.
