# Supplementary material for: Thermal Tolerance and Preferred Temperature in the Critical Endangered Montseny Brook Newt (Calotriton arnoldi)
Source: Animals (Basel). 2024 Jul 2;14(13):1963. doi: 10.3390/ani14131963 (PMC11240504; doi:10.3390/ani14131963)
Supplement: Supplementary file 1 [file animals-14-01963-s001.zip › animals-3054522-supplementary.pdf]

C.arnoldi (Western + Eastern subpopulations)			
Males			
Number	Code	weight (g)	Ct max (°C)
1	956000005590258	4,1	31,8
2	956000006040381	6,8	31,7
3	956000006105376	5,9	31,8
4	956000006085946	6,1	31,4
5	956000006109886	6	31,8
6	9560000061099043	5,3	31,6
7	956000006041005	6,7	31,6
8	956000006100855	5,7	31,8
9	956000006042725	6,1	31,6
10	956000006107043	5,7	31,8
11	956000006046381	5,5	31,8
12	956000006104107	7	31,7
	**Average**	5,908	31,70

Females			
Number	Code	weight (g)	Ct max (°C)
1	1370893341499264	7,6	31,3
2	1364137357942656	7,5	31,1
3	1405768475942784	7,4	31,5
4	1406837922799488	6,4	31,4
5	1351080657362816	6,7	31,5
6	1353301155454848	6,6	31,5
7	956000007552541	4,4	31
8	956000006040962	4,2	31,6
9	956000005677756	4	31,5
10	956000005684257	4	31,6
11	956000006039943	5,2	31,5
12	956000006090224	4,2	31,5
	**Average**	5,68	31,42

Juveniles		
Nmber	weight (g)	Ct max (°C)
1	2,3	32,1
2	2,1	31,9
3	2,1	31,4
4	3,1	31
5	2,2	31,7
6	2,2	31,2
7	2,7	31,9
8	2,3	31,8
9	2,1	31,8
10	1,8	31,8
11	2,2	31,5

12	3	30,2
**Average**	2,34	31,53

**Larvae 2017**		
**Nmber**	**weight (g)**	**Ct max (°C)**
1	0,7	33,6
2	0,9	33,6
3	0,5	33
4	0,9	33,6
5	0,7	33,6
6	0,8	33,6
7	1	32
8	1,2	31,6
9	1,7	32,2
10	1,4	31,8
11	1,5	31,1
12	1,6	30,5
**Average**	1,08	32,52

**Larvae 2018**	
**Nmber**	**Ct max (°C)**
1	32,2
2	32,2
3	32,4
4	32,3
5	32,3
6	32,3
7	31,5
8	31,5
9	31,5
10	30,9
11	30,7
12	30,5
Average	31,69
